# High Expression of KIF20A Is Associated with Poor Overall Survival and Tumor Progression in Early-Stage Cervical Squamous Cell Carcinoma

**DOI:** 10.1371/journal.pone.0167449

**Published:** 2016-12-12

**Authors:** Weijing Zhang, Weiling He, Yongjie Shi, Haifeng Gu, Min Li, Zhimin Liu, Yanling Feng, Nianzhen Zheng, Chuanmiao Xie, Yanna Zhang

**Affiliations:** 1 Sun Yat-Sen University Cancer Center, State Key Laboratory of Oncology in South China, Collaborative Innovation Center for Cancer Medicine, Guangzhou, People’s Republic of China; 2 Department of Gastrointestinal Surgery, The First Affiliated Hospital of Sun Yat-Sen University, GuangZhou, People’s Republic of China; 3 Department of Clinical Examination, The Second Affiliated Hospital of Guangzhou Medical University, Guangzhou, People’s Republic of China; 4 Eight-year program, Zhongshan Medical School, Sun Yat-Sen University, Guangzhou, People’s Republic of China; University of North Carolina at Chapel Hill School of Medicine, UNITED STATES

## Abstract

**Background:**

The kinesin family member 20a (KIF20A) protein has been implicated in the development and progression of many human cancers; however, its precise function and role in cervical cancer remain largely unclear. This study aimed to investigate the expression profile and prognostic value of KIF20A in patients with early-stage cervical squamous cell carcinoma.

**Methods:**

We examined the mRNA and protein levels of KIF20A in eight cervical cancer cell lines and eight paired cervical cancer samples, compared with normal cervical epithelial cells and adjacent normal cervical tissues, respectively. Immunohistochemistry was performed to detect the expression of KIF20A in paraffin-embedded specimens from 169 early-stage cervical squamous cell carcinoma patients. Statistical analyses were applied to analyze the association between KIF20A expression and clinical variables, as well with patient survival.

**Results:**

The mRNA and protein expression levels of KIF20A were significantly elevated in cervical cancer cell lines and lesions compared with normal cells and corresponding normal tissues (*P* < 0.05). Immunohistochemistry analysis in 169 cervical cancer cases revealed that increased KIF20A expression was strongly associated with human papillomavirus (HPV) infection (*P* = 0.008), clinical stage (*P* = 0.001), tumor recurrence (*P* = 0.016), vital status (*P* < 0.001), the property of the surgical margin (*P* = 0.032), the lymphovascular space involvement (*P* = 0.014), and pelvic lymph node metastasis (*P* = 0.001). The overall survival and disease-free survival of patients with high levels of KIF20A expression were significantly poorer than those with low KIF20A expression. KIF20A was an independent survival prognostic factor, as evidenced by univariate and multivariate analysis.

**Conclusions:**

Our results illustrate that elevated KIF20A expression correlates with HPV infection, clinical stage, tumor recurrence, lymphovascular space involvement, pelvic lymph node metastasis, and poor outcome in early-stage cervical squamous cell carcinoma patients. KIF20A aberrant expression is a novel independent unfavorable prognostic factor and may present a potential therapeutic target for cervical cancer.

## Introduction

Cervical cancer is the third most common cancer in women worldwide and accounts for the death of ~20 million women per year [1], with cervical squamous cell carcinoma (SCC) accounting for ~85–90% of all cervical cancer cases [2]. Persistent infection with high-risk human papillomavirus (HPV) types is the major causative agent of cervical cancer [3]. Standard treatments include surgical resection, chemotherapy, and radiotherapy, which are administered according to the clinical stage [5]. More recently, advances in early detection methods and preventative treatments, such as the Pap smear screening program and the HPV vaccine, have improved the prognosis of patients with cervical cancer [4]. Despite this, the clinical outcome of a large number of cervical cancer patients remains unsatisfactory as a result of tumor recurrence and metastasis [6].

Pathological factors including tumor diameter, pelvic lymph node metastasis (PLNM), lymph vascular space invasion, depth of the stromal invasion, and parametral extension have been implicated in the prognosis of cervical cancer patients [7]. While many novel oncogenes (such as *URG4*, *CISD2*, *C14ORF166*, and *B3GNT3*) are associated with the progression and prognosis of cervical cancer [8–11], they are not sufficient or accurate enough to predict patient prognosis. Thus, novel molecular biomarkers are required to predict the prognosis of patients with cervical SCC.

One potential biomarker for cervical SCC is the kinesin family member 20a (KIF20A) protein. KIF20A is 890 amino acid microtubule-associated motor protein responsible for intracellular organelle transport and cell division [12]. KIF20A, also known as RAB6KIFL/MKlp2, was first identified as localizing to the Golgi apparatus, where it was involved in the dynamics of this organelle [13]. Since then, KIF20A has been implicated in mitosis, cell adhesion, spreading, migration, and proliferation[14–21]. In addition, recent studies suggest that KIF20A is involved in tumor progression and angiogenesis [16–18,22–30].

With regards to its role in tumorigenesis, KIF20A has been shown to be essential for chromosome segregation and mitosis in breast cancer, and KIF20A expression correlates with poor disease-free survival (DFS) in patients with ER-positive breast cancer [16, 22]. Similarly, studies have implicated KIF20A expression in pancreatic cancer [17, 23], human bladder tumors [25, 26], gastric tumors [27], head and neck malignant tumors [29], and lung cancers [30]. Moreover, KIF20A contributes to both normal and pathologic hepatocyte proliferation, and is linked to tumor aggressiveness in human hepatocellular carcinomas [18]. In addition, decreased levels of endogenous KIF20A in pancreatic ductal adenocarcinoma cells altered the subcellular localization of the DLG5 protein (a scaffolding molecule involved in cell-cell contact) from the cytoplasmic membranes to the cytoplasm, resulting in drastically attenuated pancreatic cancer cell growth [24]. Furthermore, KIF20A is suggested to be a novel melanoma-associated antigen, and a potential diagnostic and prognostic marker of melanoma [28]. However, to date, few researchers have reported the role of KIF20A in cervical SCC.

The purpose of this study was to examine the expression pattern of KIF20A in cervical cell lines and human cervical SCC specimens. We further explored the association of KIF20A expression with the clinicopathological features in early-stage cervical SCC and its relationship with patient survival.

## Methods

### Ethics statement

Prior to the study, informed consent was obtained from all participants and ethical approval was obtained from the Research Ethics Committee of the Sun Yat-Sen University Cancer Center. In detail, all patients were informed that specimens which obtained from them would be used in different kinds of scientific studies in our hospital before their operations. Then they signed their written consents. Moreover, the ethics committees of Sun yat-sen University Cancer Center have approved this consent procedure.

### Cell lines

Eight human cervical cancer cell lines (HeLa, ME-180, HeLa229, SiHa, CasKi, HCC94, MS751, C33A) were provided by the ATCC Cell Biology Collection. They were maintained in RPMI-1640 medium (GIBCO BRL), which was supplemented with 10% fetal bovine serum (HyClone Laboratories, Logan, UT) and 1% antibiotics (100 U/ml penicillin and 100 μg/ml streptomycin), in the Department of Experimental Research. A primary culture of normal cervical epithelial cells was obtained from a biopsy of non-cancerous cervical epithelium and was grown in complete Keratinocyte SFM medium (Invitrogen, Carlsbad, CA, USA). Cell cultures were maintained at 37°C with 5% CO2, and the medium was renewed every 2–3 days.

### Patient and tissue specimens

Eight pairs of cervical SCC specimens and matched tumor-adjacent morphologically normal samples were obtained from the resection tissues of cervical cancer patients treated by radical hysterectomy and lymphadenectomy at the Sun Yat-Sen University Cancer Center in 2015. Patients did not receive immunotherapy, chemotherapy, or radiation before the surgery. The tissues were immediately snap-frozen in liquid nitrogen and stored at -80°C until use for investigation purposes.

In this study, a collection of 169 cervical SCC and their matched adjacent tissues were obtained from the archives of Department of Pathology of Sun Yat-Sen University Cancer Center from 2007 to 2010, and then used for immunohistochemistry (IHC) staining. Clinicopathological information assessed included: age, tumor stage, tumor size, SCC antigen (SCC-Ag), HPV infection, PLNM, tumor recurrence, vital status, differentiation grade, myometrium invasion, property of surgical margin, infiltration of parauterine organ, lymphovascular space involvement, chemotherapy, radiation and concurrent chemotherapy, and radiotherapy. They were collected from patients’ medical records and are summarized in Table 1 and S1 Table. Our hospital used polymerase chain reaction detection of HPV to determine the pre-operative high risk TYPES of human papillomavirus infection results of patients. The tests detected 13 high risk TYPES of human papillomavirus, including HPV16,18,31,33,35,39,45,51,52,56,58,59 and 68. And the normal range of the HPV testing results is from 0.00–1.00. Patients with results more than 1 will be classified as groups infected with one or more types of these 13 high-risk types of HPVs. And they were separated into group “HPV infection(+)”. Patients with results less than 1 were separated into group “HPV infection(-)”. In our study, 136 cervical cancer patients showed HPV infection(+), while 33 cervical cancer patients were HPV infection(-). The tumor stages of all the patients were defined according to the International Federation of Gynecologists and Obstetricians (FIGO) staging system [31]. Histological type and grade were diagnosed according to the World Health Organization (WHO) criteria [32]. None of the patients received chemotherapy, radiotherapy or immunotherapy before the surgery. Patients who died from unknown causes or in an emergency were excluded from this study. The 169 patients were in IB1–IIA2 stage of cervical SCC and all received a radical hysterectomy and lymphadenectomy. The median age of the cervical cancer patients was 46 years, ranging from 23 to 68 years. Patients were followed up regularly in the clinic. In accordance with our treatment guidelines in clinic, high-risk factors of cervical cancer included PLNM, positive surgical margin, positive lymphovascular space involvement, deep stromal invasion, positive parametrial involvement, high differentiation grade, and large tumor size (>4 cm). Patients with any of high-risk factors received postoperative chemotherapy and/or radiotherapy. In detail, patients with only deep stromal invasion or positive surgical margins received radiotherapy while patients with only lymphovascular space involvement, high differentiation grade, or large tumor size (>4 cm) received chemotherapy.

**Table 1:** Clinicopathological characteristics and tumor expression of KIF20A in patients with early-stage cervical squamous cell carcinoma.

Characteristic	Number of cases (%) (%)
**Age (years)**	
≤46	86 (50.9)
>46	83 (49.1)
**FIGO stage**	
IB1	74 (43.8)
IB2	25 (14.8)
IIA1	52 (30.8)
IIA2	18 (10.6)
**Tumor size (cm)**	
<4	129 (76.3)
≥4	40 (23.7)
**Squamous cell carcinoma antigen (ng/ml)**	
≤1.5	80 (47.3)
>1.5	89 (52.7)
**HPV Infection**	
No	33 (19.5)
Yes	136 (80.5)
**Pelvic lymph node metastasis**	
No	121 (71.6)
Yes	48 (28.4)
**Tumor recurrence**	
No	155 (91.7)
Yes	14 (8.3)
**Vital status (at last follow-up)**	
Alive	141 (83.4)
Dead	28 (16.6)
**Differentiation grade**	
G1	53 (31.4)
G2	101 (59.7)
G3	15 (8.9)
**Myometrium invasion**	
<1/2	64 (37.9)
≥1/2	105 (62.1)
**Property of surgical margin**	
No	156 (92.3)
Yes	13 (7.7)
**Infiltration of parauterine organ**	
No	160 (94.7)
Yes	9 (5.3)
**Lymphovascular space involvement**	
No	142 (84.0)
Yes	27 (16.0)
**Chemotherapy**	
No	82 (48.5)
Yes	87 (51.5)
**Radiotherapy**	
No	162 (95.9)
Yes	7 (4.1)
**Concurrent chemotherapy and radiotherapy**	
No	153 (90.5)
Yes	16 (9.5)
**Expression of KIF20A**	
Low or none	110 (65.1)
High	59 (34.9)

### Quantitative and real-time RT-PCR analysis

Total RNA from cultured cell lines and eight surgically dissected tumor and matched tumor-adjacent tissues was isolated using the Trizol reagent (Invitrogen, Carlsbad, CA) as per manufacturer’s instructions. Agilent Bioanalyzer 2100 was used to evaluate the RNA quality (RIN: 2.4–8.8; median 5.9). For PCR-mediated synthesis and amplification of KIF20A cDNA, an initial amplification reaction using KIF20A-specific primers was performed with a denaturation step at 95°C for 10 min, followed by 30 denaturation cycles at 95°C for 60 s, primer annealing at 55°C for 30 s, and primer extension at 72°C for 30 s. A final extension at 72°C for 5 min was carried out before the reaction was stopped and stored at 4°C on completion of the cycling steps. KIF20A-specific primers were designed using the Primer Express v 2.0 software (Applied Biosystems) as follows: forward 5’-TAACAAGGGCCTAACCCTCA-3’ and reverse 5’-TGCTCTGTCGTCTCTACCTCC-3’. To control the variability in expression levels, expression data of KIF20A were normalized to the expression of the housekeeping gene, GADPH. Primers for GADPH were 5’-TTGAGGTCAATGAAGGGGTC-3’ and 5’-GAAGGTGAAGGTCGGAGTCA-3’. Quantitative RT-PCR was performed in a total volume of 10 μl using the Light-Cycler 480 instrument (Roche Diagnostics, Penzberg, Germany) with the following conditions: 95°C for 5 min, followed by 45 cycles of 95°C for 30 s, 55°C for 30 s, and 72°C for 15 s, and a final extension step of 72°C for 5 min. The relative quantitative value was expressed by the 2-ΔΔCt method, where Ct represents the threshold cycle for each transcript. All samples were analyzed in triplicate in three independent experiments.

### Western blot analysis

Total protein from the cervical tissues and cell lines were extracted with cell lysis buffer, and the protein concentration was quantified using an Enhanced BCA Protein Assay Kit. Western blots were performed according to standard methods in our previous publication [33]. Briefly, we separated equal amounts of protein samples (30 μg) on 9% SDS polyacrylamide gels and transferred them to PVDF membranes (Immobilon P, Millipore, Bedford, MA). Anti-KIF20A rabbit polyclonal (1:1000, Thermo) and anti-Rabbit (1:2000, Santa Cruz Biotechnology, Santa Cruz, CA) antibodies were used to detect KIF20A protein. After detection, the blotted membranes were stripped, and anti-α-Tubulin was detected using a mouse monoclonal antibody (1:2000, Sigma). The secondary antibody (anti-mouse antibody, Santa Cruz Biotechnology, Santa Cruz, CA) was diluted 1:2000 in both samples. KIF20A signals were visualized on X-ray film using enhanced chemiluminescence (Amersham; Buckinghamshire, UK).

### Immunohistochemical assessment

All paraffin-embedded cervical cancer tissues and surrounding non-tumor tissues from 169 early-stage cervical SCC patients were cut into 4-μm-thick serial sections. The procedures of immunohistochemical (IHC) staining were carried out with standard protocols [33]. In detail, paraffin tissue slides (4 μm) were dried at 60°C for 1 h, deparaffinized for 10 min in xylene (twice), and rehydrated through a series of incubations in graded ethanol solutions (100%, 100%, 95%, 90%, and 80%) for 5 min, respectively. Endogenous peroxidase activity was blocked with 3% hydrogen peroxide in methanol for 15 min at room temperature to quench endogenous peroxidase activity. Following this, all deparaffinized sections were immersed and boiled in buffered ethylenediaminetetraacetic acid (pH 8.0) for 2 min in a pressure cooker to retrieve antigen, and then cooled to room temperature. After that, sections were immersed with 1% bovine serum albumin (BSA) to avoid the non-specific binding. Subsequently, the slides were immunostained with a primary antibody against KIF20A (Thermo, USA) diluted at 1:100 at 4°C overnight in a moist chamber. Phosphate buffered saline (PBS) was used as a negative control. After washing with PBS buffer, the slides were incubated with prediluted anti-rabbit secondary antibody (Abcam) and then incubated with streptavidin–horseradish-peroxidase complex (Abcam). The tissue sections were immersed in 3-amino-9-ethylcarbazole, counterstained with 10% Mayer’s haematoxylin, dehydrated and mounted in Crystal Mount. For visualization, tissue slides were stained with DAB (3,3-diaminobenzidine) for 1 min at room temperature. Finally, sections were counterstained with hematoxylin, dehydrated, and mounted.

The sections were reviewed and scored independently by two observers (pathologists) who were blinded to knowledge of the clinicopathological data. To evaluate IHC expression of KIF20A, we applied a scoring system based on multiplying the proportion of positively stained tumor cells and the intensity of staining. The staining intensity (SI) was graded according to the following criteria: 0, no staining; 1, weak staining; 2, modest staining; and 3, strong staining. The proportion of positive tumor cells was scored as follows: 0 (no positive tumor cells); 1 (<10% positive tumor cells); 2 (10–50% positive tumor cells); 3 (51–80% positive tumor cells), and 4 (>80% positive tumor cells). The slides were rescored if the difference between the two pathologist’s scores was >3. In the statistical analysis, a final staining score equal or less than 4 was used to categorize tumors with low KIF20A expression, and a score equal or more than 6 was used for high KIF20A expression.

### Statistical analysis

All calculations were carried out using SPSS version 18.0 (SPSS Inc., Chicago, IL, USA). We assessed the relationship between KIF20A expression with clinical and histomorphological characteristics by the Chi-square test or Fishers’ exact test. The overall survival (OS) and DFS curves in association with KIF20A expression were plotted using Kaplan-Meier method and the difference between the groups was tested by log-rank test. Multivariate analysis was conducted independently for each biomarker including only significant clinical/histomorphological factors from the univariate analysis. Univariate and multivariate survival analyses were conducted using the Cox regression model. All *P*-values were two-sided, and statistical significance was set at a value of *P* < 0.05.

## Results

### KIF20A is overexpressed in human cervical cancer

**Fig 1:**
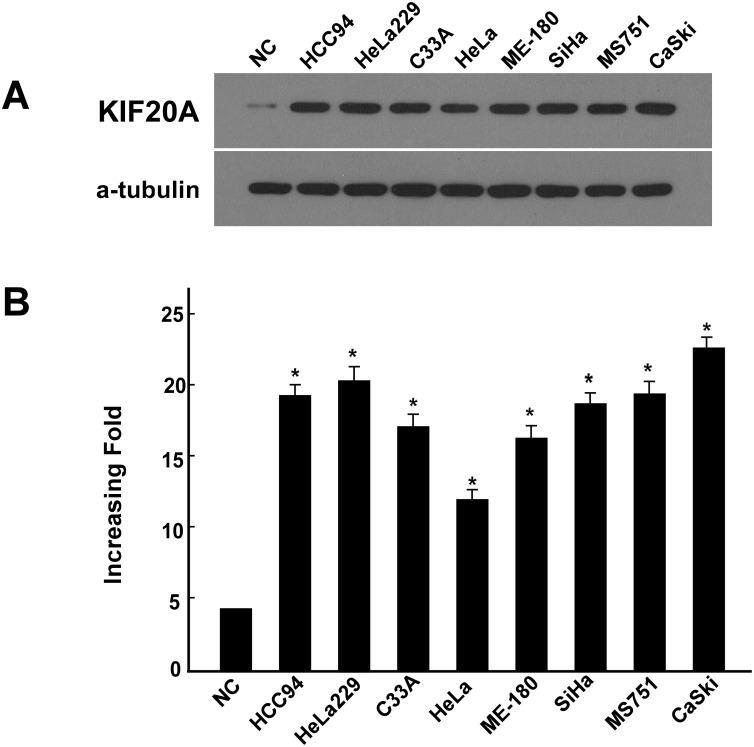
Upregulation of KIF20A mRNA and protein in cervical cancer cell lines. A, B Expression of KIF20A mRNA and protein in cervical cancer cell lines (HeLa, ME-180, HeLa229, SiHa, CasKi, HCC94, MS751, C33A) and normal cervical cell lines were examined by Western blotting (A) and qPCR (B). Expression levels were normalized against α-Tubulin and GAPDH respectively. Error bars represent the standard deviation of the mean (SD) calculated from three parallel experiments. *P < 0.05.

**Fig 2:**
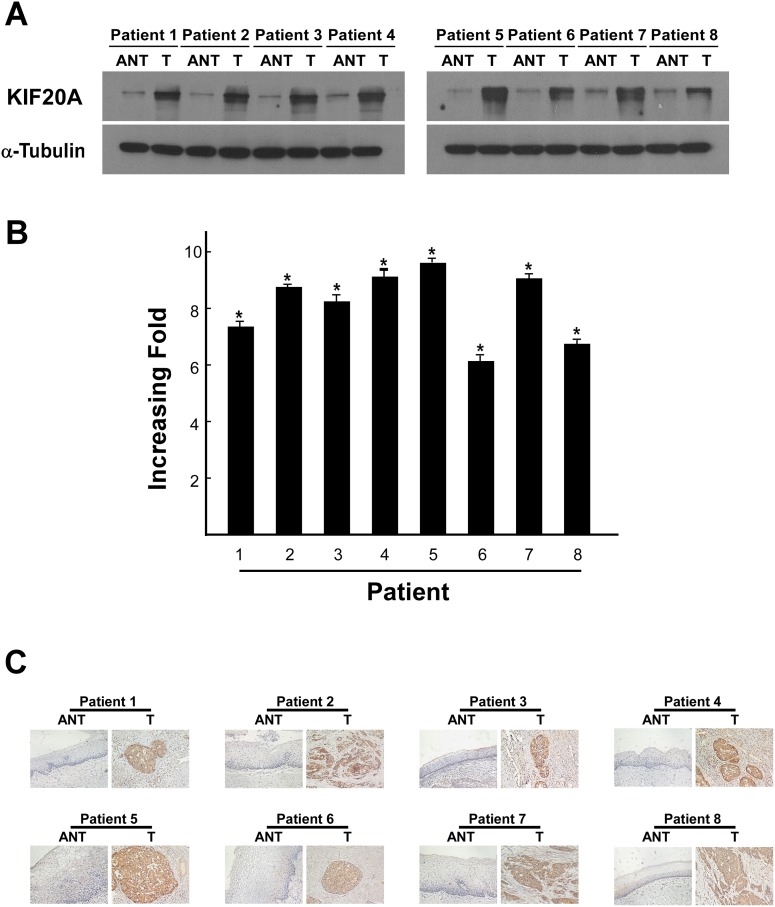
Aberrant expression of KIF20A mRNA and protein in early-stage cervical squamous cell carcinoma tissues. (A) Representative images of western blotting analyses of KIF20A protein expression in eight matched pairs of cervical cancer tissue samples (T) and adjacent noncancerous tissues (ANT). α-Tubulin was used as the loading control. (B) The average T/ANT ratios of KIF20A mRNA expression in the paired cervical cancer (T) and adjacent noncancerous tissue sections (ANT) were quantified using qPCR and normalised against GAPDH. The error bars represent the standard deviation of the mean (SD), which was calculated from three parallel experiments. (C) Immunohistochemical staining of KIF20A protein in eight pairs of matched early-stage cervical squamous cell carcinoma tissues. *P < 0.05.

All eight cervical cancer cell lines exhibited significantly elevated protein and mRNA (up to 5-fold) levels of KIF20A compared with normal cervical epithelial cells (*P* < 0.05; Fig 1). KIF20A expression was also significantly higher in the eight human cervical cancer tissues compared with the paired attached non-cancerous cervical tissues at both the transcriptional (up to 9-fold) and translational levels (*P* < 0.05; Fig 2A and 2B). In agreement with the result of the Western blot analysis and real-time RT-PCR assay, IHC analysis indicated aberrant expression of the KIF20A protein in early-stage cervical SCC lesions (Fig 2C).

### Upregulation of KIF20A is associated with clinicopathological parameters of early-stage cervical SCC

**Fig 3:**
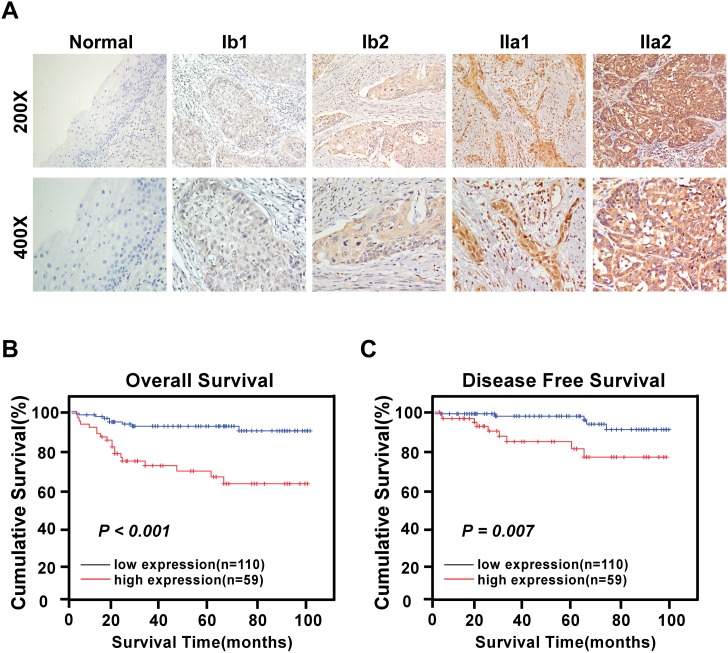
Expression of the KIF20A protein in archived paraffin-embedded early-stage cervical squamous cell carcinoma tissue sections by immunohistochemistry. A. Representative images from immunohistochemistry analyses of KIF20A expression in normal cervical epithelium tissue and different FIGO stages of early-stage cervical squamous cell carcinoma tissues. B. Patients with higher KIF20A expression in tumor were closely correlated with poorer overall survival (left) and recurrence-free survival (right) than that with tumor with lower KIF20A expression (P < 0.05, respectively).

In this study, 169 paraffin-embedded cervical early-stage SCC tissue specimens were obtained for IHC analysis. The relationships between KIF20A protein expression in cervical SCC patients and several clinicopathological variables are summarized in Tables 2 and 3. KIF20A staining was mainly observed in the cytoplasm and rarely in the nucleus of epithelial cells (Fig 3A). Following IHC staining, KIF20A expression was assessed as positive in 59/169 (34.9%) cases, and weakly positive or negative in 110 cases (65.1%). Notably, the staining of KIF20A protein in most cancerous lesions in the primary cervical tumors was statistically higher than that in the surrounding adjacent normal cervical regions. Moreover, the KIF20A protein expression was generally negative in normal cervical tissues, weak in stage IB1 and IB2 cervical SCC tissues, and strong in stage IIA1 and IIA2 cervical SCC tissues. A significant positive correlation between KIF20A protein staining and tumor grades was observed (*P* < 0.05, Fig 3A).

**Table 2:** Correlation between KIF20A protein expression and the clinicopathologic features of patients with early-stage cervical squamous cell carcinoma.

Characteristic		Total	KIF20A		Chi-squared test *P*-value	Fisher’s exact test *P*-value
			No or weak expression	Moderate or strong expression		
**Age (years)**	≤46	86	60 (35.5)	26 (15.4)	0.194	0.202
	>46	83	50 (29.6)	33 (19.5)		
**HPV infection**	No	33	28 (16.6)	5 (3.0)	0.008	0.008
	Yes	136	82 (48.5)	54 (31.9)		
**FIGO stage**	IB1	74	60 (35.5)	14 (8.3)	0.001	-
	IB2	25	16 (9.5)	9 (5.3)		
	IIA1	52	25 (14.8)	27 (16.0)		
	IIA2	18	9 (5.3)	9 (5.3)		
**Squamous cell carcinoma antigen (ng/ml)**	≤1.5	80	58 (34.3)	22 (13.0)	0.055	0.075
	>1.5	89	52 (30.8)	37 (21.9)		
**Tumor size (cm)**	<4	129	89 (52.7)	40 (23.7)	0.056	0.061
	≥4	40	21 (12.4)	19 (11.2)		
**Tumor recurrence**	No	155	105 (62.1)	50 (29.6)	0.016	0.021
	Yes	14	5 (3.0)	9 (5.3)		
**Vital status (at last follow-up)**	Alive	141	101 (59.8)	40 (23.7)	<0.001	<0.001
	Dead	28	9 (5.3)	19 (11.2)		
**Differentiation grade**	G1	53	34 (20.1)	19 (11.2)	0.445	-
	G2	101	64 (37.9)	37 (21.9)		
	G3	15	12 (7.1)	3 (1.8)		
**Chemotherapy**	No	82	53 (31.4)	29 (17.2)	0.904	1.000
	Yes	87	57 (33.7)	30 (17.7)		
**Radiotherapy**	No	162	106 (62.7)	56 (33.1)	0.652	0.696
	Yes	7	4 (2.4)	3 (1.8)		
**Concurrent chemotherapy and radiotherapy**	No	153	101 (59.8)	52 (30.8)	0.436	0.425
	Yes	16	9 (5.3)	7 (4.1)		
**Myometrium invasion**	<1/2	64	45 (26.6)	19 (11.2)	0.266	0.319
	≥1/2	105	65 (38.5)	40 (23.7)		
**Property of surgical margin**	No	156	98 (58.0)	58 (34.3)	0.032	0.035
	Yes	13	12 (7.1)	1 (0.6)		
**Infiltration of parauterine organ**	No	160	104 (61.5)	56 (33.1)	0.919	1.000
	Yes	9	6 (3.6)	3 (1.8)		
**Lymphovascular space involvement**	No	142	98 (58.0)	44 (26.0)	0.014	0.026
	Yes	27	12 (7.1)	15 (8.9)		
**Pelvic lymph node metastasis**	Absent	121	88 (52.1)	33 (19.5)	0.001	0.001
	Present	48	22 (13.0)	26 (15.4)		

**Table 3:** Correlation between KIF20A protein expression and the clinicopathological characteristics of patients with early-stage cervical squamous cell carcinoma.

Variable	KIF20A protein expression	
	Spearman’s correlation coefficient	*P*-value
**Age**	0.100	0.196
**HPV infection**	0.204	0.008
**FIGO Stage**	0.305	<0.001
**Pelvic lymph node metastasis**	0.254	0.001
**Squamous cell carcinoma antigen**	0.147	0.056
**Tumor size**	0.147	0.056
**Recurrence**	0.185	0.016
**Vital status**	0.308	<0.001
**Differentiation grade**	-0.049	0.525
**Survival time**	-0.147	0.056
**Chemotherapy**	-0.009	0.905
**Radiotherapy**	0.035	0.655
**Concurrent chemotherapy and radiotherapy**	0.060	0.439
**Myometrium invasion**	0.086	0.269
**Property of surgical margin**	-0.165	0.032
**Infiltration of parauterine organ**	-0.008	0.919
**Lymphovascular space involvement**	0.189	0.014

Chi-squared test and Fisher’s exact test were conducted to detect the associations among the protein expression levels of KIF20A protein and the clinical features of early-stage cervical SCC patients (Table 2). Analysis of the 169 early-stage cervical SCC samples indicated that KIF20A protein expression was markedly correlated with HPV infection (*P* = 0.008), FIGO stage (*P* = 0.001), tumor recurrence (*P* = 0.016), vital status (*P* < 0.001), the property of the surgical margin (*P* = 0.032), lymphovascular space involvement (*P* = 0.014), and PLNM (*P* = 0.001; Table 2). No significant difference between KIF20A protein expression and other clinicopathological features, such as age, SCC-Ag, tumor size, differentiation grade, myometrium invasion, or infiltration of the parauterine organ was observed (*P* > 0.05; Table 2). Patients with increased KIF20A protein expression showed no obvious tendency to receive chemotherapy, radiation and concurrent chemotherapy, or radiotherapy. Association coefficient analyses were performed to reveal the correlation between KIF20A protein expression levels and clinicopathological parameters, and the results were consistent with the above mentioned data (Table 3).

### Aberrant KIF20A protein expression is correlated with poor prognosis of patients with early-stage cervical SCC

We further analyzed the impact of KIF20A protein overexpression on OS. Kaplan–Meier survival analysis and log-rank test were applied to investigate whether patients with KIF20A-positive samples had a poorer survival rate than those patients with KIF20A-negative samples. Using the log-rank test, we revealed that the survival time was markedly different between these two groups. Kaplan-Meier analysis demonstrated that KIF20A-positive patients exhibited significantly reduced OS (log-rank, *P* < 0.001) and DFS (log-rank, *P* = 0.007) times than KIF20A-negative patients (Fig 3B and 3C). The cumulative 5-year survival rate was 65.2% in patients with a high level of KIF20A expression, compared with the 92.1% (*P* < 0.001) in their counterparts. These results indicate that upregulation of KIF20A protein expression was correlated with strongly poorer prognosis compared with those exhibiting low KIF20A expression (*P* < 0.001).

**Fig 4:**
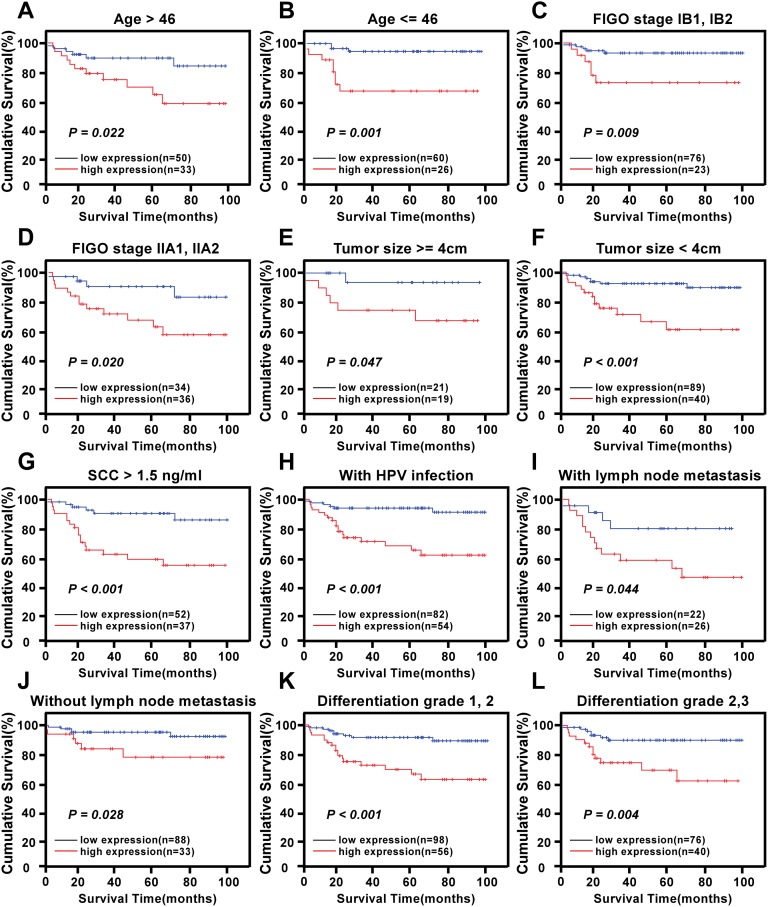
Kaplan-Meier curves of univariate analysis data (log-rank test) in select patient subgroups. Survival curves for the patients (A) with age > 46 years, (B) with age ≤ 46 years, (C) at stages IB1-IB2, (D) at stages IIA1-IIA2, (E) with tumor size ≥ 4cm, (F) with tumor size < 4cm, (G) with SCC > 1.5ng/ml, (G) with HPV infection, (H) with lymph node metastasis, (I) without lymph node metastasis, (J) at differentiation grade 1,2, (K) at differentiation grade 2,3.(P < 0.05, respectively).

**Fig 5:**
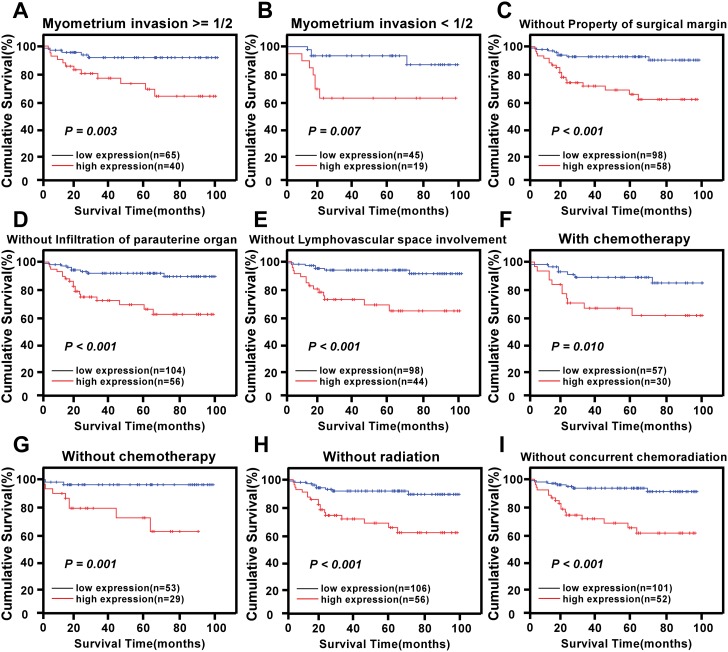
Survival curves for the patients in select patient subgroups (log-rank test). OS rates for patients (A) with myometrium ≥ 1/2, (B) with myometrium<1/2, (C) without property of surgical margin, (D) without infiltration of parauterine organ, (E) without lymphovascular space involvement, (F) with chemotherapy, (G) without chemotherapy, (H) without radiation, (I) without concurrent chemoradiation.(P < 0.05, respectively).

The prognostic value of different levels of KIF20A protein expression was also explored when stratifying the early-stage cervical SCC patients according to their age, HPV infection, FIGO stage, PLNM, SCC-Ag, tumor size, differentiation grade, chemotherapy, radiotherapy, concurrent chemotherapy and radiotherapy, myometrium invasion, the property of the surgical margin, infiltration of the parauterine organ, and lymphovascular space involvement. Expression of KIF20A protein was strongly associated with the OS duration of patients with age >46 years (log-rank test, *P* = 0.022, Fig 4A), with age ≤46 years (log-rank test, *P* = 0.001, Fig 4B), with FIGO stage IB1–IB2 (log-rank test, *P* = 0.009, Fig 4C), with FIGO stage IIA1–IIA2 (log-rank test, *P* = 0.020, Fig 4D), with tumor size ≥4 cm (log-rank test, *P* = 0.047, Fig 4E), with tumor size <4 cm (log-rank test, *P* < 0.001, Fig 4F), with SCC-Ag >1.5 ng/ml (log-rank test, *P* < 0.001, Fig 4G), with HPV infection (log-rank test, *P* < 0.001, Fig 4H), with PLNM (log-rank test, *P* = 0.044, Fig 4I), without PLNM (log-rank test, *P* = 0.028, Fig 4J), with histological differentiation grade 1–2 (log-rank test, *P* < 0.001, Fig 4K), with histological differentiation grade 2–3 (log-rank test, *P* = 0.004, Fig 4L), with myometrium invasion ≥1/2 (log-rank test, *P* = 0.003, Fig 5A), with myometrium invasion <1/2 (log-rank test, *P* = 0.007, Fig 5B), without the property of the surgical margin (log-rank test, *P* < 0.001, Fig 5C), without infiltration of the parauterine organ (log-rank test, *P* < 0.001, Fig 5D), without lymphovascular space involvement (log-rank test, *P* < 0.001, Fig 5E), with chemotherapy (log-rank test, *P* = 0.010, Fig 5F), without chemotherapy (log-rank test, *P* = 0.010, Fig 5G), without radiotherapy (log-rank test, *P* < 0.001, Fig 5H), and without concurrent chemoradiotherapy (log-rank test, *P* < 0.001, Fig 5I).

Univariate Cox regression analysis revealed that clinical variables, including upregulation of KIF20A protein (*P* < 0.001), PLNM (*P* < 0.001), SCC-Ag (*P* = 0.003), and tumor recurrence (*P* < 0.001) were significantly correlated with OS (Table 4). Following this, in the multivariate analysis, KIF20A protein overexpression (*P* = 0.032; RR = 2.557, 95% CI: 1.086–6.021), PLNM (*P* = 0.025; RR = 2.509, 95% CI: 1.124–5.601), SCC-Ag (*P* = 0.020, RR = 3.175, 95% CI: 1.199–8.408), and tumor recurrence (*P* = 0.014, RR = 2.951, 95% CI: 1.249–6.974) remained independent poor prognostic factors for OS in the early-stage cervical SCC patients (Table 4).

**Table 4:** Univariate and multivariate analyses of prognostic factors in early-stage cervical squamous cell carcinoma using a Cox-regression model.

	Univariate analysis			Multivariate analysis		
	No. of patients	*P*	Regression coefficient(SE)	*P*	Relative risk	95% confidence interval
**Pelvic lymph node metastasis**		< 0.001	1.465 (0.387)	0.025	2.509	1.124–5.601
Absent	121					
Present	48					
**Squamous cell carcinoma antigen (ng/ml)**		0.003	1.480 (0.494)	0.020	3.175	1.199–8.408
≤1.5	80					
>1.5	89					
**KIF20A**		< 0.001	1.487 (0.405)	0.032	2.557	1.086–6.021
Low expression	110					
High expression	59					
**Recurrence**		< 0.001	1.686 (0.420)	0.014	2.951	1.249–6.974
No	155					
Yes	14					

## Discussion

This is the first study to explore the KIF20A expression pattern and its association with the clinicopathological features of early-stage cervical SCC. We found that KIF20A expression is upregulated at both the transcriptional and translational levels in human cervical cancer. This upregulation was significantly correlated with the following factors in cervical cancer patients: HPV infection, FIGO stage, tumor recurrence, vital status, the property of the surgical margin, lymphovascular space involvement, and PLNM. Furthermore, survival analysis revealed that expression of the KIF20A protein was an independent novel biomarker associated with poorer survival outcomes in cervical cancer patients.

Our results are in agreement with previous studies, which demonstrate KIF20A is overexpressed at both the mRNA and protein levels in several human malignant tumors, including bladder cancer, gastric cancer, pancreatic cancer, hepatocellular cancer, head and neck cancer, lung cancer, breast cancer, and melanoma [14,16–17,19–21,23,28–30,34–35]. As the proliferation and migration of pancreatic ductal adenocarcinoma cells were significantly reduced by silencing *KIF20A* [19], KIF20A has been suggested as a novel anti-cancer drug target. Yamashita et al. suggested that immunotherapy with KIF20A may be a viable therapeutic option [28]. Indeed, KIF20A-specific Type 1 helper T cell responses have been identified in head and neck malignant tumor patients [29]. Moreover, KIF20A peptides were shown to cause expansion of HLA-A2-restricted cytotoxic T cells in HLA-A2 transgenic mice without causing autoimmunity, and those T cells successfully exhibited cytotoxic responses to cancer cells expressing KIF20A [23]. Therefore, KIF20A is a promising target for peptide-based immunotherapy for the treatment of malignancies [23].

In this study, we found that KIF20A protein expression is significantly associated with a number of advanced-disease factors, which suggests that it plays an important role in carcinogenesis and aggressiveness in cervical cancer. However, its precise mechanism of action remains unclear. KIF20A may promote the motility and invasiveness of cancer cells by transporting the RNA-binding protein IGF2BP3 and its transcripts towards the cell protrusions along microtubules, as shown in pancreatic cancer cells [17]. In breast cancer, KIF20A expression was shown to modulate mitotic spindle formation and mitotic catastrophe, which is important in paclitaxel-mediated cell death and senescence [35]. Elevated levels of KIF20A have also been correlated with ANCCA (AAA nuclear co-regulator cancer associated) levels in breast cancer tumors, whose overexpression is associated with poor patient outcomes [16]. Furthermore, knockdown of KIF20A strongly impeded proliferation and induced apoptosis of both tamoxifen-sensitive and tamoxifen-resistant breast cancer cells [16]. Another study showed that KIF20A/Mklp2 inhibition interferes with *in vitro* angiogenesis in the absence of mitosis [20]. Dysregulation of Mad2 (mitotic arrest deficient 2) was also shown to cause cytokinesis failure by misregulating KIF20A and the chromosome passenger complex, and so contributes to chromosome instability and tumorigenesis [14]. Finally, inhibition of KIF20A specifically induced potent p53-independent apoptosis at non-DNA damaging concentrations in Ewing sarcoma cell lines [21]. Therefore, KIF20A has a diverse range of functions that may together contribute to the increased tumorigenesis observed in patients with cervical SCC with high KIF20A expression. However, the precise mechanisms by which KIF20A impacts cervical cancer progression and patient prognosis requires further investigation.

In addition to KIF20A expression, we found that the SCC-Ag was an independent prognostic factor associated with poorer survival outcomes in cervical cancer patients. SCC-Ag has previously been identified as a tumor marker for the diagnosis and monitoring of cervical carcinoma [36], and radioimmunoassay for this antigen is potentially useful in clinical care [37]. Indeed, measurement of SCC-Ag levels in human body fluids (mainly in serum) was shown to be useful for diagnosis, therapy monitoring, and early recurrence detection of uterine cervical cancer [38]. It is reported that serum SCC-Ag levels are related to tumor stage, tumor size, depth of stromal invasion, lymph-vascular space status, parametrial involvement, and LNM status [39]. Indeed, SCC-Ag mRNA levels for the molecular diagnosis of sentinel LNM in cervical cancer more accurately identifies patients at risk for recurrence than the routine histology does [40]. Previous studies have elucidated that aberrant serum SCC-Ag can precede the clinical diagnosis of relapse in 46–92% of cases [41]. Preoperative levels of SCC-Ag are also useful for predicting the status of post-surgical high-risk factors in women with SCC of the uterine cervix who undergo radical hysterectomy [42]. Other studies indicate that the serum SCC-Ag marker is not useful for early detection for cervical cancer but is useful indicator for advanced stage and prognosis of the disease [43]. They can also be used to guide appropriate treatment choice; for example, elevated pretreatment levels of SCC-Ag indicate a poor response to neoadjuvant chemotherapy [44] and can detect disease recurrence in patients treated with concurrent chemoradiotherapy [45]. In keeping with these previous observations, our study demonstrated that serum SCC-Ag level is an independent prognostic biomarker in early-stage cervical SCC. Nonetheless, it still remains debatable whether serum SCC-Ag levels may represent a prognostic variable and be useful for earlier diagnosis of relapse [46, 47]. Other studies have found that SCC-Ag level is not an outstanding predictive biomarker for pelvic and paraaortic LNM [48]. Recently, several reports raised the issue that the cutoff levels for SCC-Ag employed in various studies are different, thus affecting the sensitivity and specificity of prediction [49]. Therefore, it is necessary to explore biomarkers other than SCC-Ag to predict clinical outcomes in patients with cervical SCC. Our findings proved that KIF20A was an independent prognostic factor associated with poorer survival outcomes in cervical SCC patients. Whether KIF20A is better than SCC-Ag to predict clinical outcomes in patients with cervical SCC needs further studies.

Our present study also showed that elevation of KIF20A protein expression is markedly correlated with HPV infection. Persistent infection (i.e., 10 to 20 years) with high-risk HPV types can progress to cervical cancer [50, 51]. In particular, the *E6* and *E7* HPV genes are known to be oncogenic [52]. These E6/E7 oncoproteins have been shown to upregulate A3B (a major mutagenic protein) expression in HPV-induced tumorigenesis [53]. Other proteins, such as KLF13 and DGCR8, have also been implicated in HPV-induced tumorigenesis [54, 55]. In this study, aberrant KIF20A protein expression was observed in HPV-positive cervical cancer samples (31.9%), whereas HPV-negative specimens expressed low levels of KIF20A (16.6%). Moreover, the expression of KIF20A protein was significantly associated with the OS duration of patients with HPV infection. Our results suggest that KIF20A might make the cervix more prone to HPV infection, which provides insight into the HPV-induced carcinogenesis process. Nevertheless, more extensive work needs to be carried out to determine the molecular mechanisms of the association between KIF20A expression and HPV infection.

PLNM has also been shown to be an independent prognostic parameter in cervical cancer [56]. The overall 5-year survival rate of early-stage cervical cancer patients without PLNM is 81%, while in those with PLNM it is reduced to 53% [57]. PLNM is also useful for determining the optimal postoperative therapy (e.g., after radical hysterectomy plus lymphadenectomy, patients with PLNM should receive postoperative chemotherapy and radiation). Unfortunately, current clinical examinations (such as computed tomography or magnetic resonance imaging) show low accuracy [58], and in developing countries (such as China), the increasing costs and waiting time of these clinical examinations might delay diagnosis and treatment. Therefore, molecular markers of PLNM in cervical cancer are urgently required. The current data suggest that PLNM may act as an independent indicator of poor clinical outcomes in early-stage cervical SCC patients. In addition, high level expression of the KIF20A protein was strongly correlated with PLNM. Interestingly, KIF20A protein overexpression can serve as a good prognostic marker of poor postoperative OS for cervical cancer patients with a positive pelvic lymph node status. These demonstrations imply that KIF20A expression may help us to identify patients with PLNM, and that these patients should undergo more aggressive therapy in order to reduce cancer mortality. However, a detailed understanding of the mechanisms of KIF20A in regulating PLNM of early-stage cervical cancer is still necessary.

While the present study proved the prognostic significance of KIF20A protein expression in patients with early-stage SCC, there were some limitations, including its small sample size. Therefore, further studies with a larger sample size are needed to validate our results. It is interesting to detect the expression pattern of serum KIF20A protein or mRNA expression levels and analyze the relationship between it and clinicopathological data. Moreover, functional analysis of KIF20A protein modification during tumor proliferation and metastasis is required to provide information on its functional significance in the progression of cervical cancer.

## Conclusions

In conclusion, our study suggests that the expression levels of KIF20A contribute to progression of cervical cancer. KIF20A protein expression in early-stage cervical squamous cell cancer was significantly associated with aggressive clinicopathological features and it is a good predictor for HPV infection, FIGO stage, lymphovascular space involvement, and pelvic lymph node metastasis. Furthermore, KIF20A protein was identified as an independent marker for predicting the clinical outcome of early-stage cervical squamous cell cancer patients.

## Supporting Information

S1 Table The basic clinical characteristics of all samples. (XLS)
